# En Bloc Resection With Reconstruction Using a Customized Megaprosthesis in a Case of Proximal Humerus Giant Cell Tumor: A Case Report

**DOI:** 10.7759/cureus.34217

**Published:** 2023-01-25

**Authors:** Mihir H Maniar, Sushil Mankar, Rahul Sakhre

**Affiliations:** 1 Orthopedics and Traumatology, NKP Salve Institute of Medical Sciences & Research Centre, Nagpur, IND

**Keywords:** case report, megaprosthesis, en-bloc resection, proximal humerus, giant cell tumor

## Abstract

A giant cell tumor is a common, benign but locally aggressive bone tumor faced by orthopedic surgeons. The proximal humerus is a rare site of occurrence for this tumor, and the challenges posed while approaching such a case are discussed in this report of a 29-year-old male who presented with pain, swelling, and restricted motion at the left shoulder. Plain radiographs and MRI were suggestive of an aggressive giant cell tumor of the proximal humerus, which was confirmed on histopathological examination. Due to the lesion's extensive soft-tissue involvement, en-bloc resection with reconstruction was planned, but due to the COVID-19 pandemic, surgery was delayed. During the same period, the patient had trivial trauma to the same shoulder, following which the size of the lesion began increasing. The patient was operated on with en-bloc resection and reconstruction with a custom megaprosthesis; following the surgery, there was a complete resolution of pain and improvement in the range of motion. En bloc resection and replacement with a customized megaprosthesis, though technically demanding, offer a safe and cost-effective modality for limb salvage surgery for large giant cell tumors, with good functional outcomes and decreased chances of recurrence.

## Introduction

A giant cell tumor (GCT) is a frequently encountered bone tumor of benign and aggressive nature that occurs in patients aged 20-45 years, occurring slightly more frequently in females [1,2]. The proximal humerus is affected in 4% of giant cell tumor cases [3]. Pain and swelling, along with restriction of shoulder movements, are the main reasons for a hospital visit in cases of proximal humerus GCT [3-5]. To treat cases of aggressive tumors, recurrences, or cases with malignant transformations, surgery is the mainstay, whereas radiotherapy is done only in cases where the tumor is inaccessible [2]. Adjunctive medical therapy with denosumab has been reported to be associated with surgical downstaging of the tumor [6]. There is controversy regarding surgical treatment because, while it is important to ensure adequate removal of tumor tissue, it is likewise crucial to retain limb function. Extended curettage gives good results in cases of well-contained GCT, but tumors with cortical breaches or those with large soft tissue extension need en bloc resection with reconstruction of the defect with an endoprosthesis to regain joint and limb function.

## Case presentation

Patient information

A 29-year-old right-handed Indian male factory worker presented with a year-long history of dull aching pain and a gradually increasing swelling over the left shoulder. Apart from these, the patient had no other complaints and did not have any history of trauma at the time of the index visit. The patient had no addictions, and neither had any contributing familial or past history. The patient was evaluated with plain radiographs, and further investigations were planned, but given the various restrictions due to the COVID pandemic, investigations and management were deferred at the time. During the same four-month period, the patient had a history of trivial trauma due to a fall over the left shoulder from standing height, following which the patient had aggravation of pain and swelling and further restriction in movement.

Clinical findings

**Figure 1:**
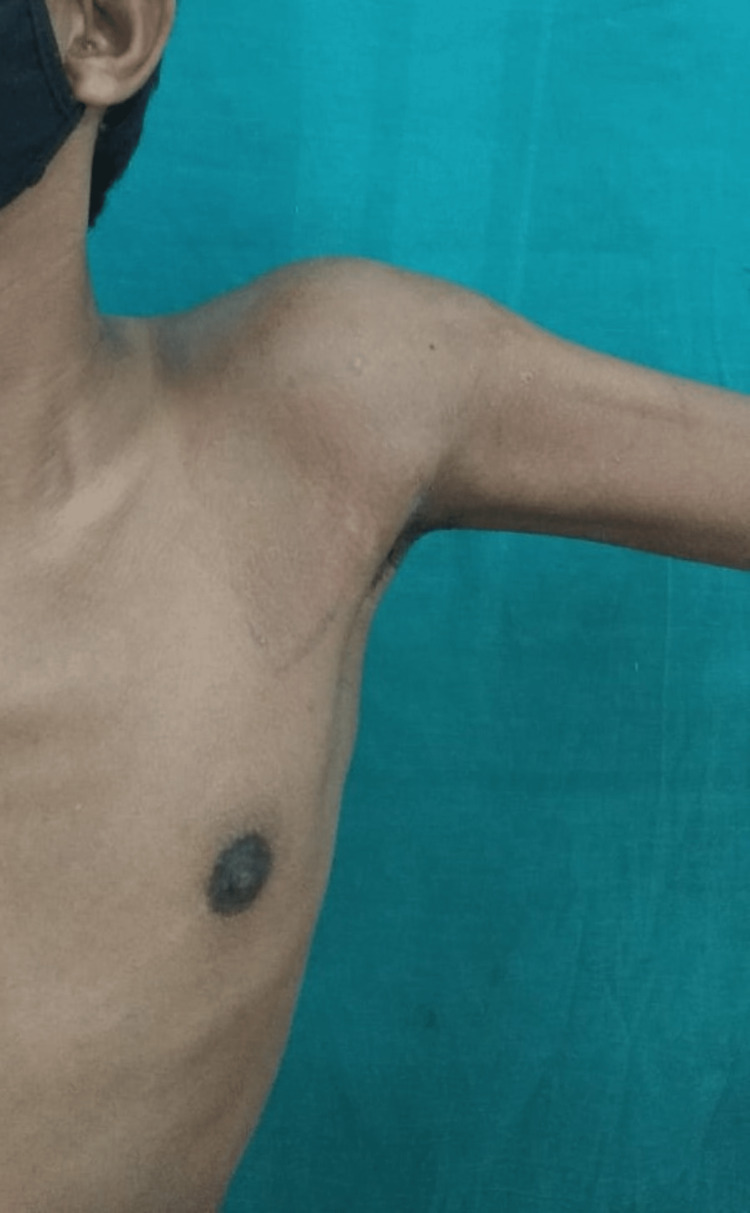
Maximum abduction possible at the index visit

The patient had a swelling over the proximal aspect of the left arm; the swelling had a smooth contour and was immobile and firm in consistency. Restricted abduction up to 80° and flexion up to 70° on examination were noted at the index visit (Figure 1). The skin over the swelling was mobile and not fixed to the underlying tissues, with no sinuses, dilated veins, or scars (Figure 1). Lymph nodes were not palpable.

**Figure 2:**
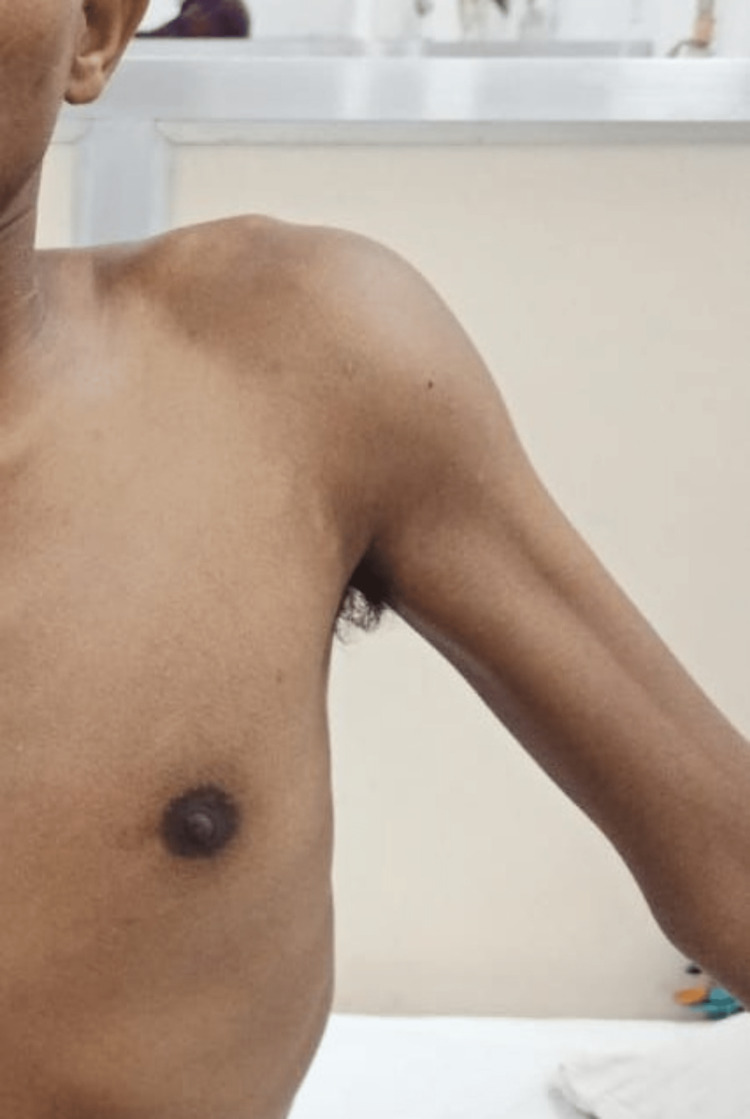
Maximum possible abduction on the second visit with an increase in the size of the swelling

On a subsequent visit four months later following the trauma, there was an increase in the size of the swelling with increased pain and tenderness and further restriction of movements, with only up to 30° of abduction and 20° of flexion and no extension possible (Figure 2).

Timeline

The patient developed a dull, aching pain over the left shoulder of insidious onset that steadily progressed over six months. The patient also noticed a swelling over the left shoulder that gradually increased in size. After eight months, the patient started experiencing difficulty carrying out daily activities, such as lifting objects overhead with the left upper limb. The patient presented at this stage with his complaints and was evaluated with plain radiographs. On suspicion of GCT, further investigations were intended, but a coinciding surge in the number of coronavirus cases in the region delayed the same due to logistical drawbacks, and the patient was lost to follow-up. For four months, there was no medical intervention except occasional analgesics that were taken by the patient himself. At this point, the patient had fallen from a standing height with impact over his left arm and presented again, this time with an aggravation of pain and further limitation of movements. The patient was now evaluated with repeat radiographs and MRI imaging. A definitive diagnosis of GCT was established using a core biopsy. A surgical plan was drawn up for en bloc resection and reconstruction. The proximal humerus was templated, and a customized proximal humerus megaprosthesis was manufactured. Surgery was carried out as per the plan; no significant unanticipated complications were encountered. On discharge, the patient had relief from pain. On one-month and three-month follow-ups, the patient reported relief from pain and partial recovery in the range of movements in the left shoulder.

Diagnostic assessment

**Figure 3:**
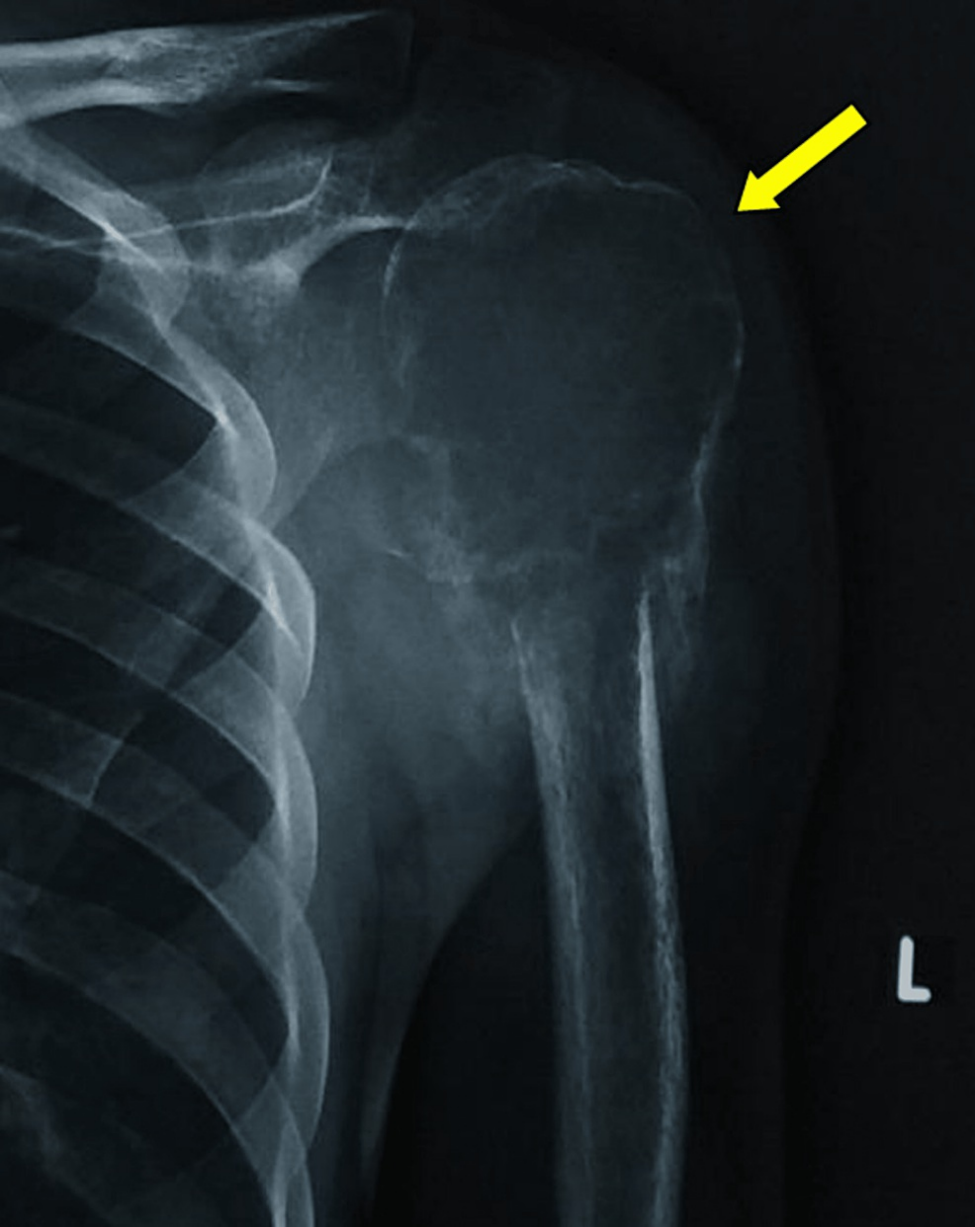
A plain radiograph at the index visit

**Figure 4:**
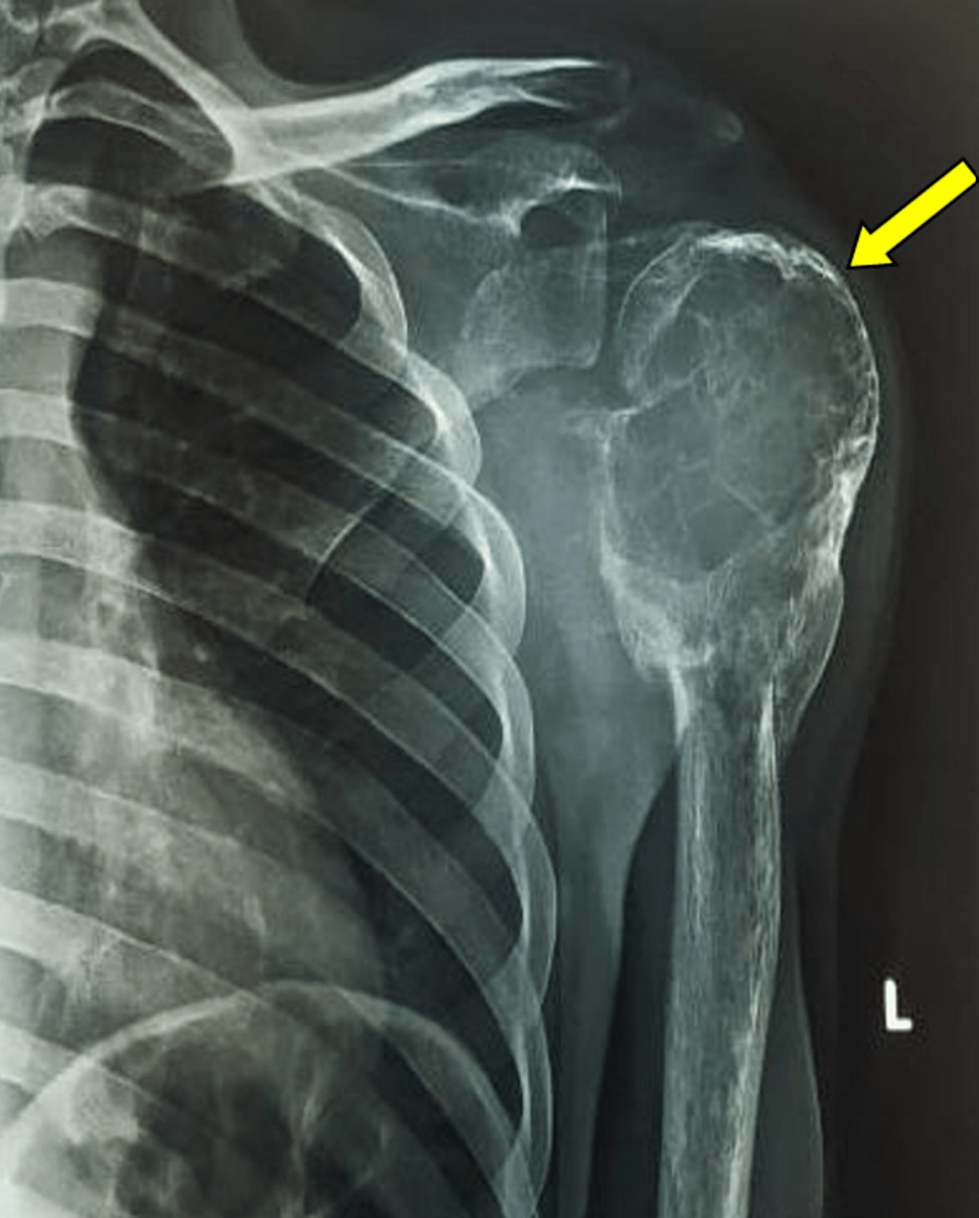
A plain radiograph on the second visit showed an increase in the size of the lesion

The evaluation was done using a plain radiograph anteroposterior view of the left shoulder, which revealed an epiphyseal-metaphyseal expanding lesion at the proximal humerus with a thin rim of cortical bone and the lesion extending to the subchondral bone. Multiple septa were seen traversing the lesion, and a characteristic soap bubble appearance was noted (Figure 3). Further investigations and management were delayed owing to the COVID pandemic. On subsequent presentation, a repeat radiographic study showed a notable enlargement in the size of the lesion as compared to that of the index visit, along with a pathological fracture that was also seen during the index visit at the diaphyseal end of the tumor (Figure 4).

 

**Figure 5:**
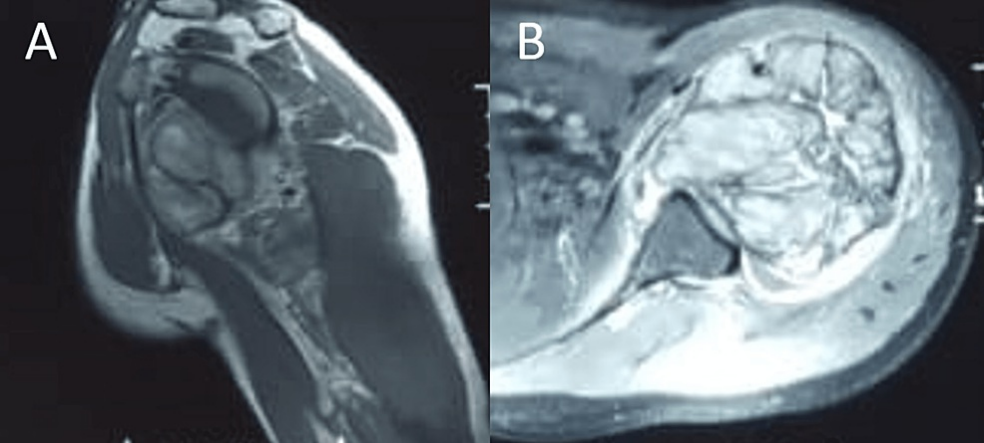
An MRI showed multiple cortical breaches and soft tissue extension; the articular surface was breached with tumor tissue eroding the articular cartilage A: coronal image; B: axial image

High suspicion of GCT on plain radiographs was further validated on an MRI, which showed multiple cortical breaches and soft tissue extension; the articular surface was breached with tumor tissue eroding the articular cartilage (Figures 5A-5B).

**Figure 6:**
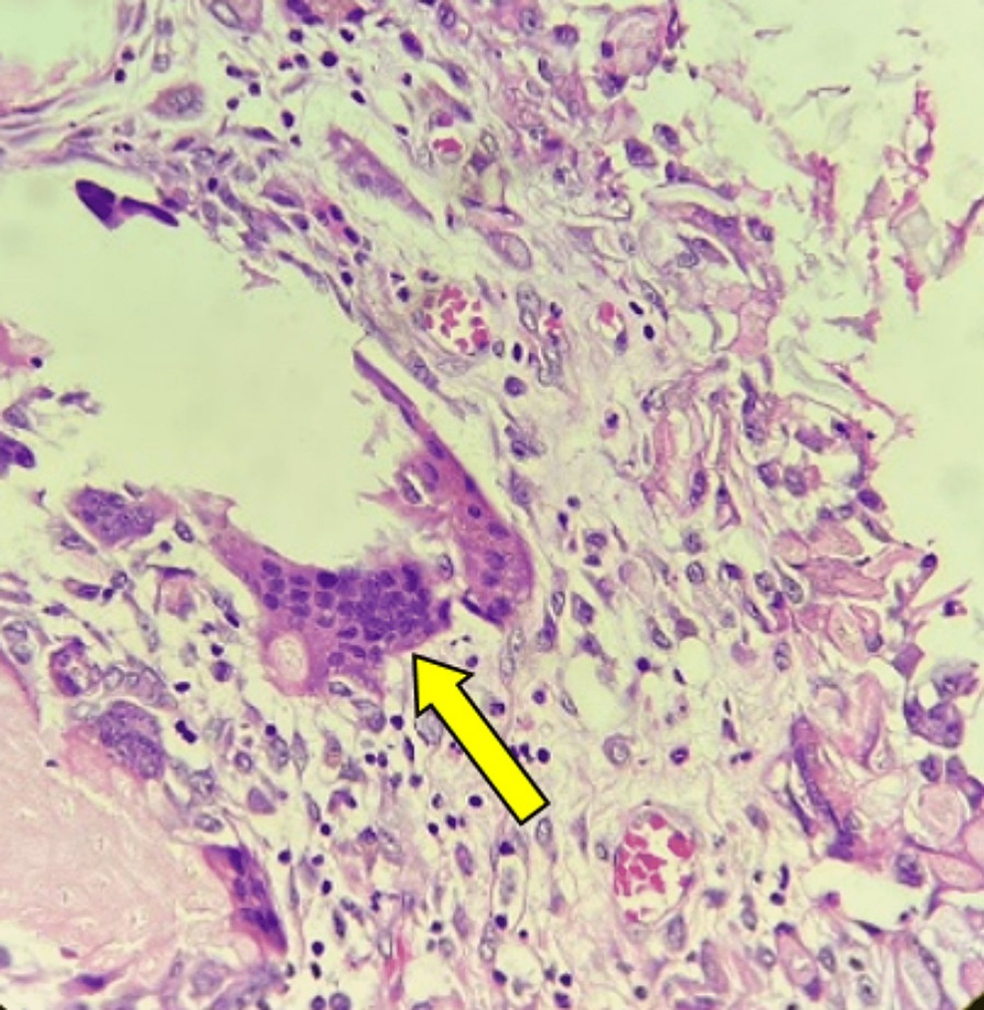
Histopathology examination Large multinucleated osteoclast-like cells (yellow arrow)

Routine blood investigations were carried out and showed normal blood counts, kidney function, and liver function. When available, it is preferable to do a PET scan in cases where limb salvage is planned; in this case, due to the unavailability of PET facilities, it could not be performed. Instead, a plain chest radiograph and high-resolution computed tomography (HRCT) were done to rule out metastatic disease. A core biopsy was performed for a histopathological diagnosis, and a giant cell tumor was confirmed (Figure 6).

Intervention

**Figure 7:**
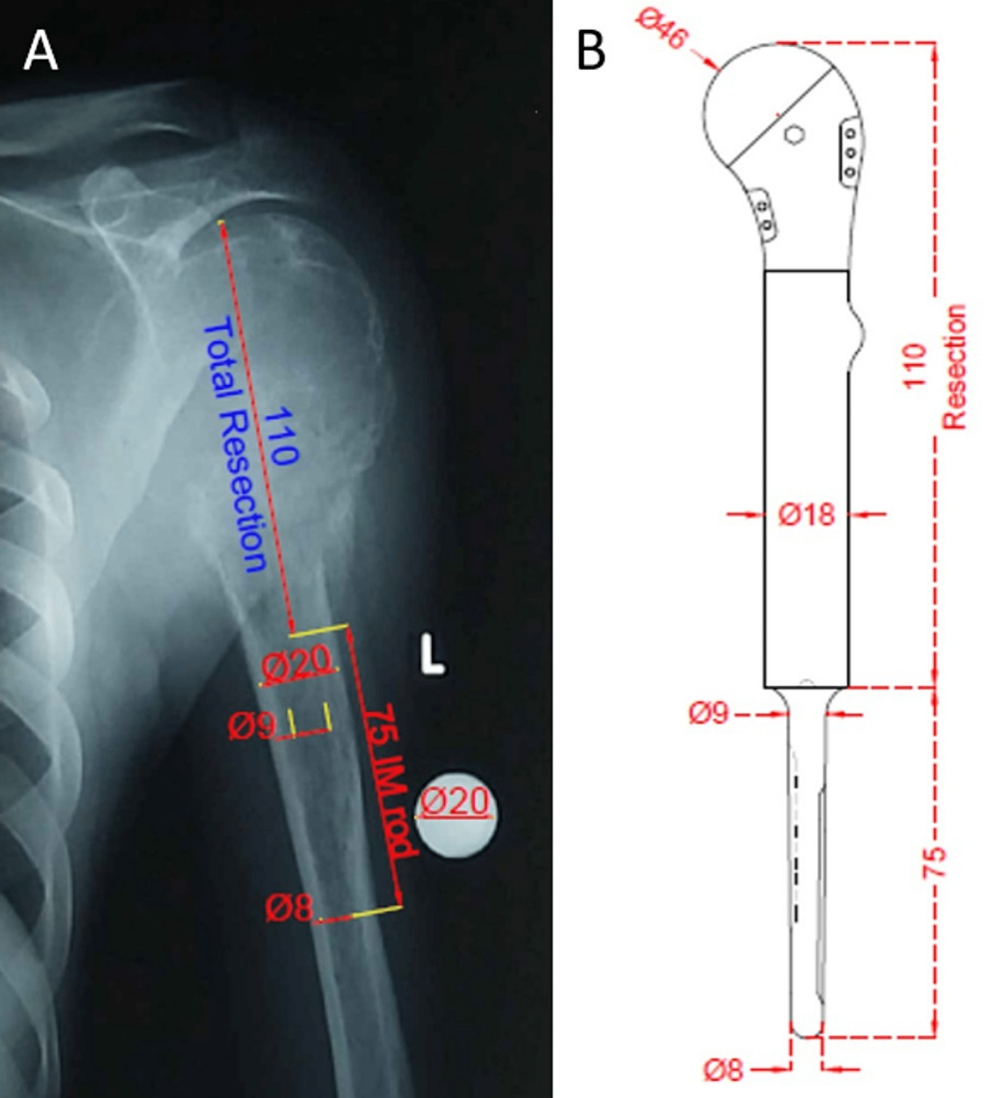
Preoperative templating for customizing the megaprosthesis

Given the extent of soft tissue involvement, en bloc resection followed by reconstruction was planned. Preoperative planning was carried out, and templating for customizing the megaprosthesis was done (Figure 7).

**Figure 8:**
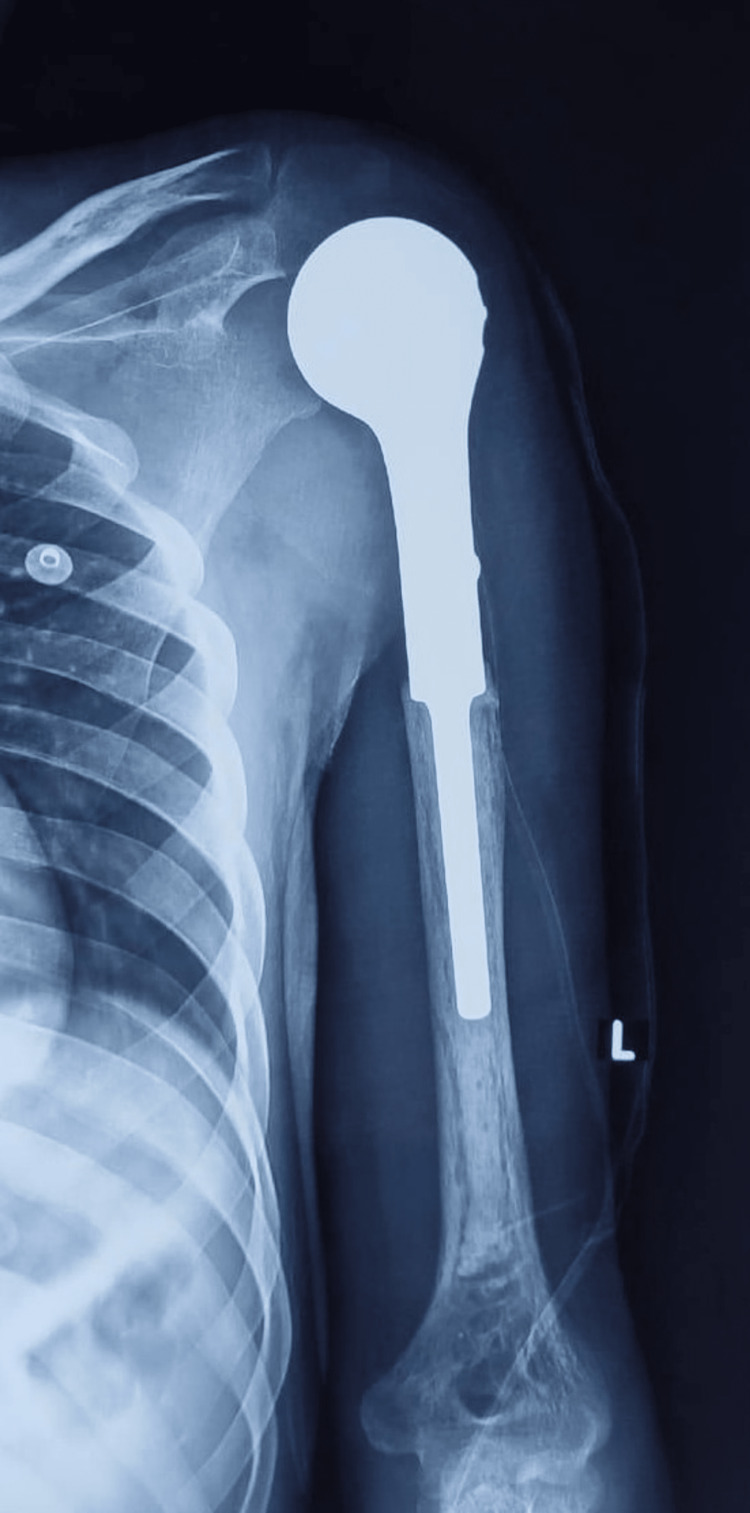
Postoperative X-ray with the customized prosthesis in situ

The deltopectoral approach was used, extending the incision from the coracoid process down to the midshaft region; major neurovascular structures were identified and protected to ensure non-contamination of the surrounding normal tissue; after exposure of the tumor, surrounding tissue was diligently packed to avoid spillover of tumor cells; and osteotomy was performed through normal bone with a distal bony margin of 1 cm. Tumor tissue was grayish-brown and soft in consistency. Resection was grossly guided until all abnormal tissue was removed and further carried out for a 1 cm margin in the normal tissue. Following the completion of curettage, a thorough saline wash was given. Thinning of the rotator cuff tendons was noted, likely attributable to disuse atrophy, which made identification of individual tendons difficult. Involved soft tissues were carefully resected, followed by a saline wash to curtail residue from resection. Reconstruction was performed by using the customized megaprosthesis with a cemented stem (Figure 8), and rotator cuff muscles were passed through the slot designed in the proximal part of the prosthesis and reflected and sutured on themselves. Postoperatively, the patient was given an abduction brace, and range of motion exercises were started gradually, first passively and then with assisted movements.

Follow-up

The patient in this case was reviewed for up to three months. Review at one month showed an improvement in the range of movement up to 35° abduction and 30° flexion, and at three months, there was an improvement in the range of movement up to 40° abduction and 40° flexion without any distal extremity functional deficit. The patient had complete relief of pain, and no surgical site complications were evident. A three-monthly follow-up routine was advised to the patient to watch for any recurrence.

## Discussion

Patients affected by GCT are young, with the most common presentation being complaints of pain and swelling. Pathological fracture is found in 10-15% of cases [5]. Giant cell tumors, despite being benign, are locally aggressive, causing destruction of the bony cortex as well as extending into the soft tissue compartment. Due to their periarticular location, if left unchecked, they may even destroy the articular surfaces. The clinical approach includes a relevant history and examination. Investigations include plain radiographs as an initial screening modality. As per anatomy, MRI is of particular use as it can delineate both intramedullary and soft tissue extension, which is difficult to ascertain on plain radiographs. However, cortical continuity is best seen on a CT scan. It is paramount in all suspected cases to look for other aggressive tumors that may have metastasized, and this is done by a PET scan, chest radiograph, and bone scan, where possible. A biopsy helps confirm the histopathological diagnosis. Either a core biopsy or an open biopsy can be performed. In such sizeable lesions, the risk of insufficient sample collection is lessened, and with improvements in histopathology techniques, a core biopsy may be preferable, as it is safer, easier to perform, more cost-effective, and gives a good representative sample of the relevant tissue with minimal trauma and minimal biopsy track seeding. The approach to treating these patients has given rise to constant debates in the field of ortho-oncology, with the aim of striking a balance between maintaining limb function and lowering the chances of recurrence. GCT has a 25% risk of recurrence [7]. A higher rate of recurrence has been seen after curettage-only surgeries; hence, isolated curettage is not performed and is rather extended with the use of adjuvant therapy that can target residual tumor cells. These include the use of a high-speed burr for its mechanical action of removing the residual cells; polymethyl methacrylate cement or cryotherapy with liquid nitrogen, which is thermal cauterization; and chemical cauterization using phenol or alcohol, with or without the use of a bone graft [8]. This line of treatment is possible when the tumor is localized with a relatively good cortical rim and >5 mm of subchondral bone. Whereas, in selected cases with extensive bone destruction and/or articular involvement, amputation may be indicated. Resecting a major proximal joint and at the same time preserving the function of the limb is a surgical challenge. Options to perform reconstruction of the proximal humerus include either glenoid-preserving hemiarthroplasty or total shoulder replacement if there is glenoid surface erosion. The use of medical therapy in the form of denosumab, when feasible, has been shown to aid in reducing areas of cortical breach and also aid in curtailing surgical time and morbidity [6]. Disarticulation is undesirable for GCT involving major proximal joints, as it would result in loss of limb and lead to a decline in quality of life. The choice of the implant varies based on numerous factors, such as the amount of bone loss after the resection and the condition of the soft tissues around the joint. The use of a customized megaprosthesis for reconstruction gives the advantage of replacing the lost bone segment while also preserving joint motion and distal limb function in cases where salvage surgeries are the only option. En bloc resection with reconstruction using megaprostheses is advantageous in that they are affordable, allow for early mobilization, and are associated with the least chances of recurrence along with a cosmetically acceptable appearance. This method of reconstruction has been reported in the literature and is considered a safe technique with good outcomes and fewer complications. En-bloc resection thus offers a safer modality to reduce recurrence rates in Campanacci grade III lesions for preventing recurrence and preserving joint function [9,10]. There have been discussions in the literature regarding the mode of restoration of the proximal humerus following tumor excision using autogenous grafts, osteoarticular allografts, prosthetic replacement with a composite allograft-prosthesis, and an autograft prosthetic composite [11-18]. Intra-operative complications of such wide resections include neurovascular injuries, significant blood loss, flap necrosis, infection, rotator cuff tears, and muscle injuries. Prosthetic loosening, breakage, or periprosthetic fractures make salvage surgeries a high-risk undertaking. There is a significant loss of dexterity immediately following the surgery, with some restrictions persisting for prolonged periods, which are attributable to extensive soft tissue release and a poor bed for soft tissue reattachment.

## Conclusions

When available, it is preferable to do a PET scan in cases where salvage is planned. A biopsy helps confirm the histopathological diagnosis. Either a core biopsy or an open biopsy can be performed. In such sizeable lesions, the risk of insufficient sample collection is lessened, and with improvements in histopathology techniques, a core biopsy may be preferable, as it is safer, easier to perform, more cost-effective, and gives a good representative sample of the relevant tissue with minimal trauma and minimal biopsy track seeding.

In this case, due to the unavailability of PET scan facilities, it was not performed, and a core biopsy confirmed the diagnosis. As the lesion was extensive on the index visit, the approach would have been along the same lines even if the pandemic had not delayed the treatment, with wide resection and endoprosthetic replacement. To ensure non-contamination of the surrounding normal tissue, after exposure of the tumor, the surrounding tissue must be diligently packed to avoid spillover of tumor cells, and following completion of curettage, a thorough saline wash must be given. Enbloc resection must be done with a 1-cm-wide margin of normal bone to ensure complete removal of abnormal tissue. Regular clinical and radiological follow-up every three months to look for any recurrence is advisable, and in case of relapse, repeat resection may be attempted. Thus, a safe, realistic route for treatment of proximal humerus GCT is with custom endoprosthetic replacement following tumor resection, which offers low rates of complication. An additional advantage is an immediate stability, which allows for aggressive recovery of movements at the shoulder joint, which in turn expedites limb function.
